# Development of a millimetrically scaled biodiesel transesterification device that relies on droplet-based co-axial fluidics

**DOI:** 10.1038/srep29288

**Published:** 2016-07-18

**Authors:** S. I. Yeh, Y. C. Huang, C. H. Cheng, C. M. Cheng, J. T. Yang

**Affiliations:** 1 Department of Mechanical Engineering, National Taiwan University, No. 1, Sec. 4, Roosevelt Road, Taipei, Taiwan; 2 Institute of Biomedical Engineering, National Tsing Hua University, No.101, Sec. 2, Guangfu Road, Hsinchu, Taiwan

## Abstract

In this study, we investigated a fluidic system that adheres to new concepts of energy production. To improve efficiency, cost, and ease of manufacture, a millimetrically scaled device that employs a droplet-based co-axial fluidic system was devised to complete alkali-catalyzed transesterification for biodiesel production. The large surface-to-volume ratio of the droplet-based system, and the internal circulation induced inside the moving droplets, significantly enhanced the reaction rate of immiscible liquids used here – soybean oil and methanol. This device also decreased the molar ratio between methanol and oil to near the stoichiometric coefficients of a balanced chemical equation, which enhanced the total biodiesel volume produced, and decreased the costs of purification and recovery of excess methanol. In this work, the droplet-based co-axial fluidic system performed better than other methods of continuous-flow production. We achieved an efficiency that is much greater than that of reported systems. This study demonstrated the high potential of droplet-based fluidic chips for energy production. The small energy consumption and low cost of the highly purified biodiesel transesterification system described conforms to the requirements of distributed energy (inexpensive production on a moderate scale) in the world.

Biodiesel, a fatty acid methyl ester (FAME), is a diesel fuel alternative to petroleum diesel that is produced from biomass resources such as vegetable oil, animal fats, waste cooking oil, and microalgae oil. Because biodiesel is renewable, biodegradable, non-toxic and environmentally beneficial^1^,^2^, it has been widely used to decrease greenhouse gas emissions and mitigate the problem of global warming. Biodiesel is generated through transesterification, which involves a reaction between triglycerides and alcohol in the presence of a chemical catalyst.

The simplest method to produce biodiesel involves the use of a batch-stirred tank reactor (BSTR)^3^,^4^,^5^ for transesterification of biodiesel with a base catalyst. The duration of the entire reaction in such a reactor generally requires a few hours. The immiscibility of oil and methanol limits the reaction rate in the production of biodiesel. In the past decade, reactors and production methods investigated to enhance the efficiency of biodiesel synthesis include a supercritical fluidic system^6^, microwave heating^7^, and an ultrasonic reactor^8^. Manufacturing biodiesel with supercritical methanol shortened the total reaction time from that of a traditional BSTR. Microwave heating provided advantages when applied in a continuous system for biodiesel production because it produced large FAME yields. Use of an ultrasonic reactor increased the material interface area between the alcohol and oil to enhance the conversion of oil through ultrasonic emulsification. Because these methods involve large operating costs and energy consumption, the development of new techniques employing efficient heat/mass transfer and increased reaction rate, such as can be achieved with microfluidics, arouses considerable attention^9^,^10^.

In recent years, microfluidic devices have been developed for biodiesel transesterification to include capillary reactors^11^,^12^ and microreactors^13^,^14^,^15^,^16^. However, a droplet-based microfluidic system with a large surface-to-volume ratio is more suitable for biodiesel transesterification because it can increase the chemical reaction rate by increasing the material interface between reagents^17^,^18^. Regarding recent two-phase immiscible reagent system reaction reaseach^19^,^20^, it has been shown that mass transfer across the boundary between two immiscible liquids in a microchannel is enhanced significantly by internal circulation in the segmented liquid^21^,^22^. Several researchers have investigated rapid mixing or a reaction in droplet-based microfluidic devices using the internal circulation flow within the droplet^23^,^24^. The two-phase flow of immiscible fluids in microchannels has considerable potential for highly efficient production in chemical engineering applications^25^.

For commercialization and mass production of biodiesel, a millimetrically scaled fluidic system was used as a compromise proposal^26^. This approach retained the advantages of a microfluidic system–e.g., rapid reaction–and mitigated the small output disadvantages of a microsystem. In this work, we designed a millimetrically scaled, droplet-based, co-axial fluidic system for biodiesel transesterification with large oil conversion and small energy consumption. In this device, the soybean oil accurately cut off the methanol droplet at a constant frequency. As a methanol droplet was surrounded by soybean oil, it decreased the molar ratio between methanol and oil to near the equivalent stoichiometric coefficients of a balanced equation (methanol/oil = 3/1), which decreased the cost and waste of methanol. Use of this system for biodiesel production entails a new approach in bioenergy research and conforms to the global requirements of distributed energy (inexpensive production on a moderate scale).

## Results

**Figure 1:**
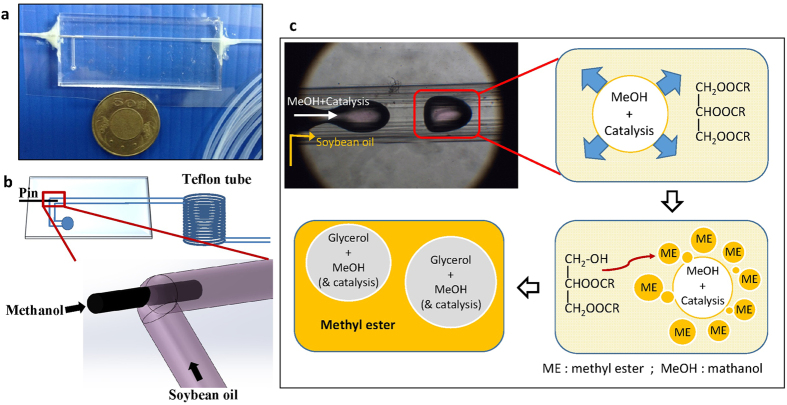
Millimetrically scaled droplet-based co-axial fluidic system: (**a**) photograph of the device, (**b**) schematic diagram of the inlet of co-axial flow, (**c**) sequential reaction schemes for transesterification in droplet-based fluidic devices. The methanol droplet was surrounded by soybean oil flow; the transesterification occurred at the interface between these two immiscible fluids. The soybean oil reacted with the methanol and was converted into glyceride and biodiesel. The glyceride was then released to the methanol phase and dissolved in residual methanol; the biodiesel was in the continuous phase.

In this study, we designed a millimetrically scaled droplet-based co-axial fluidic system for biodiesel transesterification. Figure 1a,b display a photograph and a schematic diagram of this device. Figure 1c shows the reaction scheme for transesterification in our droplet-based fluidic device.

### Droplet formation and system stability

**Figure 2:**
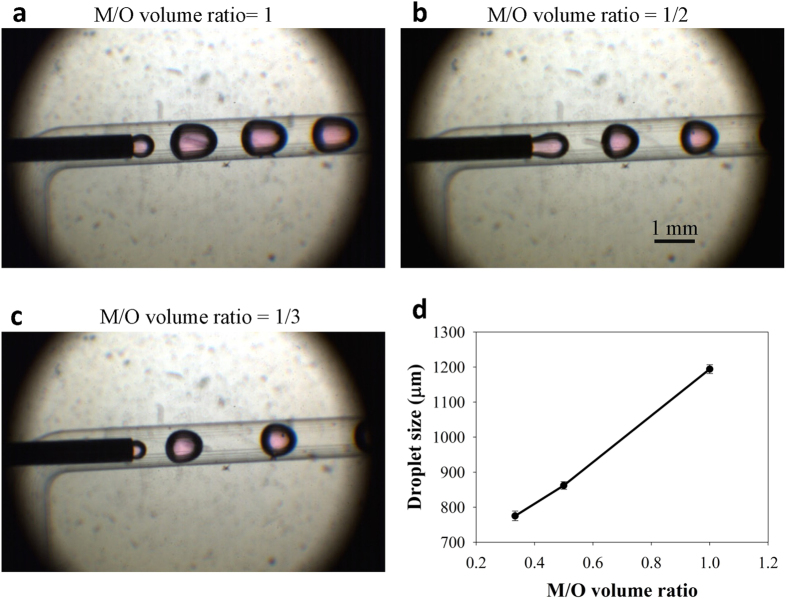
Images of methanol droplets in the co-axial flow device. M/O volume ratio was controlled to be (**a**) 1, (**b**) 1/2, (**c**) 1/3. (**d**) The linear relationship between droplet size and M/O volume ratio. Because the length of the minor axes in each droplet were almost the same as the width of the channel (1 mm), the droplet size was defined as the length of major axes of the droplet.

In this device, the methanol droplet jetted from the inner channel was cut off accurately by the soybean oil stream at a constant frequency. Figure 2a–c provide images of the methanol droplets in this co-axial flow device with varied M/O volume ratio. The methanol droplets were stable in this device when the M/O volume ratio was even less than 1/3 (M/O molar ratio < 7.9), which means that the molar ratio in the flow channel approached the stoichiometric molar ratio according to an equivalent chemical equation (M/O molar ratio = 3). The droplet size (the length of major axes of the droplet) decreased linearly with a decrease in volume ratio (see Fig. 2d).

If we seek to diminish the waste of excess methanol, the molar ratio must decrease and approach the stoichiometric coefficients of a balanced equation. For a droplet-based fluidic system, the volume ratio affected the droplet size and the frequency of the droplet generation. The material interface between two reactants was the key factor that influenced the reaction rate and was inversely proportional to the droplet size of the droplet-based fluidic system. Upon decreasing the molar ratio, the ratio of surface-to-volume of one droplet was increased and the total material interface between two reactants decreased. In this manner, optimized operating parameters of biodiesel transesterification were adopted to increase productivity and maximize the beneficial results of this device.

### Effect of temperature on biodiesel transesterification

**Figure 3:**
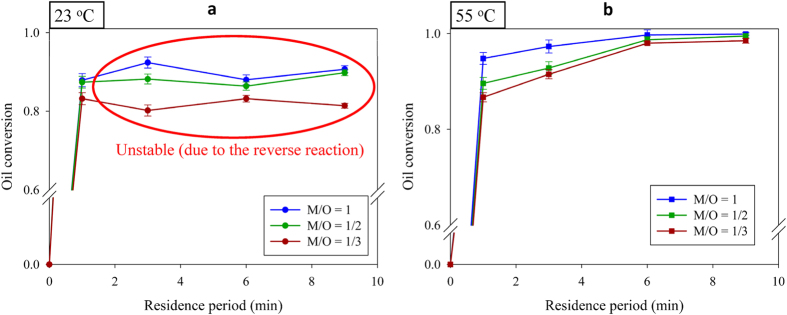
Variation of oil conversion with residence period at varied volume ratio and temperature: (**a**) 23 °C, and (**b**) 55 °C. The concentration of catalyst is 1.0 *wt*%. The reverse reaction accelerated after the oil conversion exceeded 80% at 23 °C.

A high reaction temperature increases the cost of producing biodiesel. Several microreactors have performed high-yield rapid biodiesel transesterification, but the reaction temperature exceeded 56 °C for all reported microreactors^11^,^12^,^13^,^14^,^15^,^16^. Figure 3 shows the volume variation for conversion of oil over time (residence period) at 23 °C and 55 °C. At 23 °C (Fig. 3a), the conversion exceeded 80% within 1 minute under all conditions. The forward reaction became slower and the reverse reaction become quicker after the oil conversion exceeded 80% at 23 °C; hence, oil conversion did not significantly increase and the reaction reached and maintained kinetic equilibrium even after prolonging the residence period to more than 9 minutes. The limit of biodiesel transesterification was about 80–90% at 23 °C. The forward rate constant of transesterification increased with an increase in temperature^27^, as discovered when executing the reaction at 55 °C (Fig. 3b). At this temperature oil conversion exceeded 98.5% with varied volume ratio at the 9-minute residence period. Although decreasing the excess methanol (M/O volume ratio = 1/3; M/O molar ratio = 7.9) decreased the oil conversion at the initial reaction stage (<6 minutes), the conversion maintained 98.6% after 9 minutes. The oil conversions were 100%, 99.5% and 98.6% at volume ratios = 1, 1/2, and 1/3 respectively, at the 9-minute residence period.

**Figure 4:**
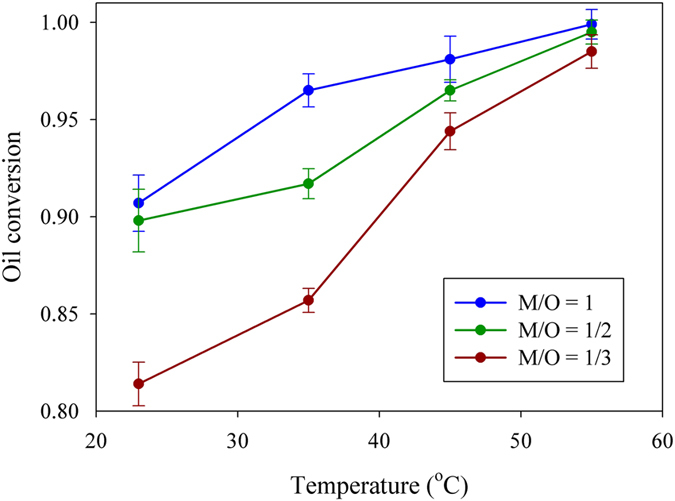
Oil conversion variation with temperature and M/O volume ratio. The concentration of catalyst is 1.0 *wt*%; the residence period is 9 minutes.

Figure 4 shows the variation of oil conversion with temperature. The concentration of catalyst is 1.0 *wt*%; the residence period is 9 minutes. At M/O volume ratio = 1, which implies much excess methanol, the conversion of oil rapidly increased when reaction temperature increased from 23 °C to 35 °C. When we decreased methanol volume, rapid growth in oil conversion occurred between 35 °C and 45 °C. With M/O volume ratio = 1/3, the oil conversion increased significantly from 85.7% to 94.7% as the temperature was increased from 35 °C to 45 °C. In a droplet-based fluidic system, increasing the reaction temperature increased not only the fraction of activated molecules but also the circulation around the moving droplet as the viscosity of the soybean oil decreased. Increasing the circulation around the moving droplet enhanced the mass transfer between the two immiscible liquids. The rate of biodiesel transesterification was accelerated both chemically and physically, which is a more effective approach than a traditional stirred batch reactor or a continuous-flow microreactor.

### Effect of catalytic concentration on biodiesel transesterification

**Figure 5:**
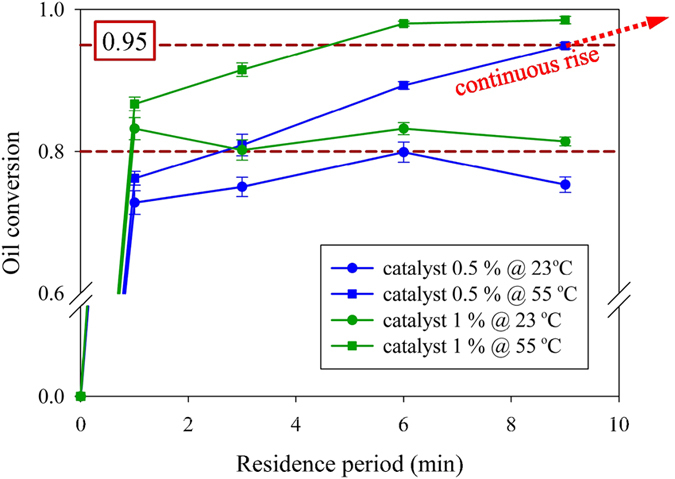
Variation of oil conversion with residence period at different catalyst concentration (0.5 and 1 *wt*%) and temperature (23 °C and 55° C). The M/O volume ratio = 1/3.

Several biodiesel transesterification microreactors^11^,^12^,^13^,^14^,^15^,^16^ used alkaline catalyst concentrations that ranged from 0.5 to 4.5 *wt*%. A greater concentration of catalyst improved the rate of biodiesel transesterification, but saponification was prone to occur when the free fatty acids or water were contained in the raw material; this saponification decreased the rate of conversion and blocked the device channels. Alkaline catalyst concentration is suggested to be typically less than 1.2 *wt*% NaOH in a microreactor^14^. As mentioned above, the conversion in our device exceeded 98% with 1.0 *wt*% of NaOH catalyst, M/O volume ratio = 1/3 and a reaction temperature of 55 °C. Even though no saponification occurred in the reaction, much water was required in the purification process to remove the residual alkali catalyst. We thus sought to decrease the concentration of catalyst in this device. The variation of oil conversion with residence period at different catalyst concentration (0.5 and 1 *wt*%) and temperature (23 °C and 55 °C) is shown in Fig. 5 (M/O volume ratio = 1/3). At the same operating temperature, the conversion of oil in the reaction with concentration 1.0 *wt*% of catalyst is approximately 5–15% greater than the conversion with 0.5 *wt*%. The trend in variation of oil conversion with residence period is almost the same at 23 °C with varied catalyst concentration, including a rapid initial growth and reversal of reaction. When M/O volume ratio = 1/3 (less excess methanol), the oil conversion saturated at 98.6% after a residence period of 6 minutes with a catalyst concentration of 1.0 *wt*%. The conversion increased linearly to 94.9% at 9 minutes and tended to rise continuously after 9 minutes with a catalyst concentration of 0.5 *wt*%. These results show that a decreased catalyst concentration required an additional 1–2 minute residence period to attain oil conversion of 98%. Using this millimetrically scaled, droplet-based, co-axial fluidic system to produce biodiesel, we must balance the energy consumption and reaction duration integrated with a purification system.

## Discussion

Table 1 presents a comparison of biodiesel transesterification methods, including two production methods on a commercial scale and one millimetrically scaled reactor. The droplet-based co-axial fluidic system has greater conversion with shorter residence period and less energy consumption than current industrial biodiesel production methods. The batch-stirred reactor requires a residence period of 3–5 hours at 65 °C for production that consumed much energy. The supercritical alcohol method requires heating and a pressurized system to maintain the operating temperature 300–350 °C and pressure of 46–65 MPa during the entire process. With our device, the energy consumption of biodiesel transesterification, the pumping of fluids, and the heating of device, uses 0.95 kJ/kg, which is much less than the energy consumption for traditional production methods on a commercial scale (typically more than 100 kJ/kg)^28^. Although several researchers have shown large conversion of oil with a brief residence period using microreactors, the output of a microreactor is insufficient to meet the real-life requirements of the energy field. In this work, we found that a millimetrically scaled system, with production scaled in liters, fulfilled the requirements of distributed energy.

A microtube reactor, which has dimensions similar to those of our system, has been used to perform biodiesel transesterification^26^; the oil conversion and operating conditions of this microtube reactor are shown in Table 1. To compare our results with those of the microtube reactor, we applied the same operating conditions (A–D) for biodiesel transesterification using our droplet-based co-axial fluidic system, which is also shown in Table 1. Alkaline catalysts are commonly used in biodiesel transesterification to dehydrate methanol through the catalysis of hydroxide ion (OH-) from a strong base. The concentration of hydroxide ion in 4.5 *wt*% KOH solution is the same as in 3.2 *wt*% NaOH solution. With the same M/O molar ratio and the same concentration of catalyst (conditions A and B), our droplet-based system showed a greater conversion of oil than was achievable with the microtube reactor using the same residence period at a reaction temperature of 60 °C. Because of the effect of the reverse reaction, the large M/O molar ratio, which provides much excess methanol, was used in a microtube reactor at 20 °C, but the conversion in that microtube reactor was less than 80% after 4 minutes. In our droplet-based system, the oil conversion was more than 92% at the same M/O molar ratio with a smaller concentration of catalyst for the same residence period at 23 °C (conditions C and D).

Because the viscosity of methanol is less than that of soybean oil, a strong internal circulation is readily generated in a methanol droplet. Circulation inside the droplet promoted the mass transfer around the boundary of the droplet that resulted in significantly increased mixing efficiency between the two reactants, thus increasing the conversion of oil in this system. The droplet-based co-axial fluidic system has better performance than other methods of continuous production. Several parameters–including operating temperature, M/O molar ratio, catalytic concentration–affected the efficiency of the conversion of oil in biodiesel transesterification. The optimized operating conditions, which consider energy consumption in the entire process and the efficiency of oil conversion, depend on the regional structure of energy resources and the integration of back-end purification systems. In this work, we introduced a droplet-based fluid system concept for biodiesel transesterification that possesses a greater rate of reaction throughput and a smaller consumption of energy and material than present production methods.

## Conclusions

We executed transesterification using an alkali catalyst at 23 °C to 55 °C and pressure at 1 bar within a droplet-based fluidic system. The transesterification involved the reaction of triglyceride lipids (soybean oil) with alcohol (methanol) to produce biodiesel. The conversion of soybean oil to biodiesel was analysed with NMR spectra. The large surface-to-volume ratio of our droplet-based device and the internal circulation induced inside the moving droplets significantly enhanced the rate of reaction in the proposed system.

Our millimetrically scaled, droplet-based co-axial fluidic system generated a methanol droplet surrounded by soybean oil, which decreased the molar ratio between methanol and oil to near the stoichiometric coefficient of a balanced equation (methanol/oil = 3/1) upon increasing the total volume of oil. The oil conversion attained over 80% at 23 °C after a residence period of 9 minutes, when the molar ratio between methanol and oil was 7.9 (M/O volume ratio = 1/3), the catalytic concentration was 1.0 *wt*%. Because the reverse reaction accelerated when the oil conversion exceeded 80% without heating, the limit of transesterification of biodiesel was about 80–90% near 23 °C. The oil conversions were 100%, 99.5%, and 98.6% at M/O volume ratios of 1, 1/2, and 1/3, respectively, and a residence period of 9 minutes at 55 °C. In a droplet-based fluidic system, an increased reaction temperature increases not only the fraction of activated molecules but also the circulation around the moving droplet as a result of the decreased viscosity of soybean oil. The rate of biodiesel transesterification was accelerated with both chemical and physical means, making this process more effective than traditional stirred-batch reactors or continuous-flow microreactors.

In this work, a droplet-based co-axial fluidic system performed better than other methods of continuous-flow production. We achieved an efficiency much greater than that of reported systems. Our experimental results demonstrate that the concept of using a millimetrically scaled, droplet-based fluid system for biodiesel transesterification requires a decreased period of reaction, has increased efficiency, and possesses great potential for biodiesel mass production. The production of this continuous production system is at least 1.2 litters per chip, per day. The production of the proposed device is sufficient for the general energy needs of family or even a small community, which is very difficult to achieve using a microreactor. This concept of biodiesel production and the design of the fluidic system are also convenient for household use such as for the conversion of cooking oil to biodiesel. Further, it is applicable in developing countries that lack financial and natural resources. This concept also conforms to the requirements of distributed energy (inexpensive production on a moderate scale), which is the global trend for green energy development.

## Methods

### Design and fabrication

The fluidic channel was fabricated through replica molding. We applied polydimethylsiloxane (PDMS) onto a polymethyl-methacrylate (PMMA) mold, which was fabricated with computer-numerical-control (CNC) machining. We placed a steel pin (24 G × 25.4 mm, 0.55 mm × 25 mm, TERUMO Corp.) into two unbonded PDMS slabs, and bonded them with an oxygen-plasma method. The diameter of the outer flow channel was 1 mm; the diameter of the inner flow channel was 0.55 mm. We varied the lengths of Teflon tube to investigate the influence of residence period on the conversion of oil under varied operating parameters including temperature and catalyst concentration. In this work, a jet of methanol flowed into the inner channel and was broken up by a stream of soybean oil that was pumped in through the side channel.

### Chemicals and analysis

The components of the soybean oil (purchased from a local market) were fatty acids palmitic acid 7.4%, oleic acid 27%, stearic acid 3.5%, linoleic acid 56.2%, linolenic acid 2.2%, and icosanoic acid 0.3%. The mean molar mass of soybean oil is 879.4 g. The acid and soap saponification values determined by standard titration methods were 0.37 mg KOH/g and 188.1 mg KOH/g, respectively. The components of fatty acid are triglycerides 97%, diglycerides 1% and monoglycerides 0.7%. We added sodium hydroxide (NaOH, anhydrous, Fisher) to dehydrated methanol (Sigma-Aldrich) to act as a catalyst to enhance the rate of biodiesel transesterification. The concentration of NaOH was calculated according to the mass ratio (relative to soybean oil). Phloxine B (Sigma-Aldrich) added to the methanol to facilitate observation acted as an unreactive red dye; it is soluble in glycerol but insoluble in oil.

Transesterification occurred at the interface between the two immiscible fluids, i.e., methanol and oil. Triglyceride, the main component of soybean oil, reacted with the methanol phase and was progressively converted into glyceride and methyl ester (as biodiesel). The glyceride, a by-product of transesterification, was released into the methanol phase and dissolved in the residual methanol. Because the methyl ester (biodiesel) and the soybean oil are miscible, the oil phase was composed of soybean oil and biodiesel in varied proportions. Transesterification was halted when the soybean oil was consumed. We collected the continuous phase to analyse the oil conversion rate of transesterification.

The oil conversion rate was analyzed with proton nuclear-magnetic- resonance spectra (^1^H-NMR, Bruker AVIII 500 MHz FT-NMR); the sampling method is shown in the supplementary documents. The average sampling error is 1.4% in this device. In biodiesel transesterification the oil conversion rate is defined as the ratio between α-CH_2_ (methylene) protons and triglyceride ester (glyceryl) protons^29^:


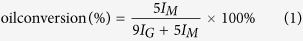


The chemical shifts are 3.6–3.7 ppm for the methyl group (*I*_*M*_) in the methyl ester and 4.1–4.3 ppm for the methylene protons (*I*_*G*_) in soybean oil.

### Experimental setup

The soybean oil and the methanol were impelled with two syringe pumps (PHD 2000, Harvard Apparatus, USA), 20-mL syringes, and tubing (Teflon, outer diameter 1.37 mm, inner diameter 1.07 mm). The excess methanol (typically 8–30 times than soybean oil) accelerated the transesterification reaction. In this work, the total flow rate in the channel was 1.2 mL/min, and the ratios between the methanol and soybean oil pumping flow rates were 1.00, 0.50, and 0.33; the molar ratios between methanol and triglyceride were 23.70, 11.85, and 7.90, respectively. In transesterification, the stoichiometric coefficients in a balanced equation for methanol and oil are 3 and 1, respectively. For stably generated droplets flow, the maximum flow rate in the channel was 1.8 mL/min; the production in this device was more than 50 mL per hour.

## Additional Information

**How to cite this article**: Yeh, S. I. *et al*. Development of a millimetrically scaled biodiesel transesterification device that relies on droplet-based co-axial fluidics. *Sci. Rep.* **6**, 29288; doi: 10.1038/srep29288 (2016).

## Supplementary Material

Supplementary Information

## Figures and Tables

**Table 1:** Comparison of different methods of biodiesel transesterification, including two production methods on a commercial scale and a millimetrically scaled reactor.

**Scale**	**Method**		**Temperature /°C**	**Molar ratio M/O**	**Concentration of catalyst/%**	**Residence period**	**Oil conversion/%**
Commercial-scale production methods	Batch-stirred reactor		65	9–30	KOH 2%	3–5 h	99
	Supercritical system– continuous-flow production		300	40	×	22 min	80
Millimetrically scaled reactor	Microtube reactor^26^	A	60	4.6	KOH 4.5	1 min	55
		B	60	4.6	KOH 4.5	4 min	80
		C	20	23.9	KOH 4.5	1 min	30
		D	20	23.9	KOH 4.5	4 min	80
	Droplet-based co-axial fluidic system (this work)	A	60	4.6	NaOH 3.2	1 min	62.9
		B	60	4.6	NaOH 3.2	4 min	88.1
		C	20	23.9	NaOH 2.2	1 min	87.9
		D	20	23.9	NaOH 2.2	3 min	92.4
