# Supplementary material for: Development of a millimetrically scaled biodiesel transesterification device that relies on droplet-based co-axial fluidics
Source: Sci Rep. 2016 Jul 18;6:29288. doi: 10.1038/srep29288 (PMC4947928; doi:10.1038/srep29288)

Development of a millimetrically scaled biodiesel transesterification device that relies on droplet-based co-axial fluidics

S. I. Yeh, Y. C. Huang, C. H. Cheng, C. M. Cheng,* and J. T. Yang,*

The conversion of biodiesel was analyzed with proton nuclear-magnetic-resonance spectra (1H-NMR, Bruker AVIII 500 MHz FT-NMR)

1. Sampling method

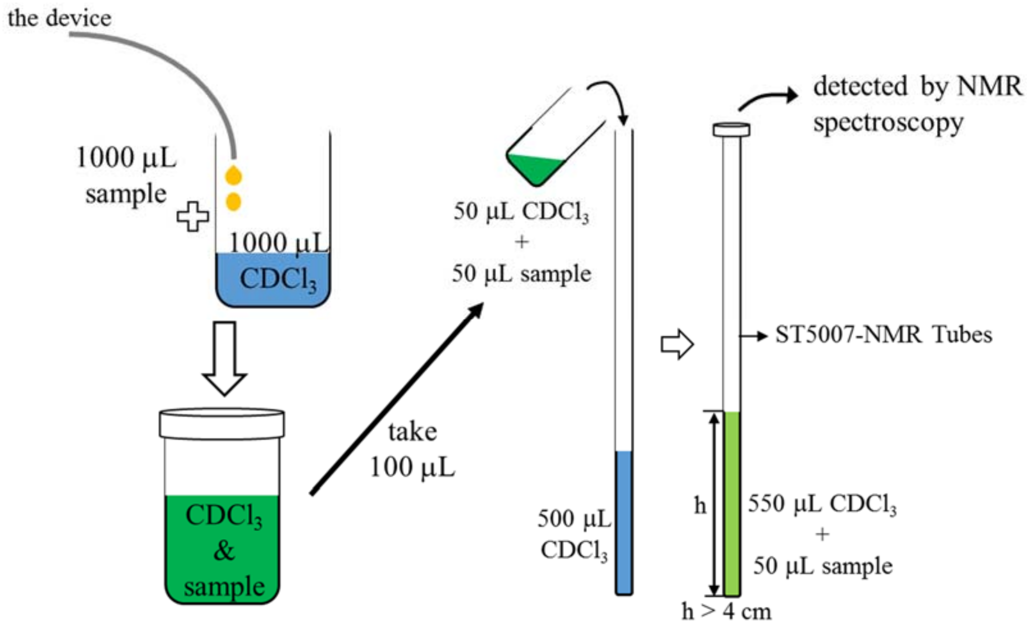

2. Spectrum (before and after transesterification)

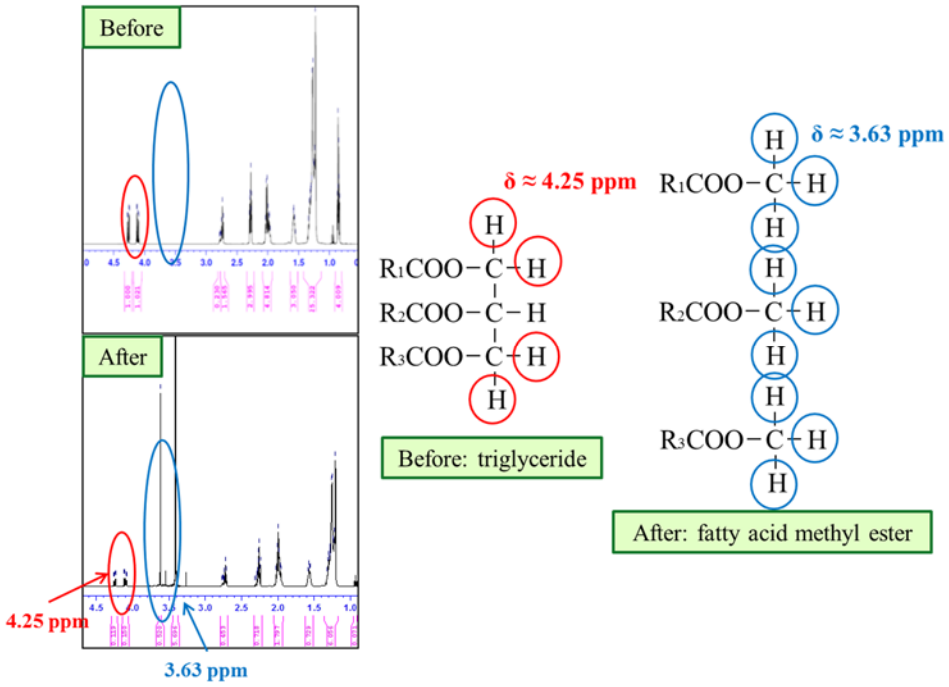

Supplement: Supplementary Information [file srep29288-s1.pdf]
